# Genetic engineering of non-native hosts for 1-butanol production and its challenges: a review

**DOI:** 10.1186/s12934-020-01337-w

**Published:** 2020-03-27

**Authors:** Said Nawab, Ning Wang, Xiaoyan Ma, Yi-Xin Huo

**Affiliations:** 1 Key Laboratory of Molecular Medicine and Biotherapy, School of Life Science, Beijing Institute of Technology, 5 South Zhongguancun Street, Haidian District, Beijing, 100081 People’s Republic of China; 2 Biology Institute, Shandong Province Key Laboratory for Biosensors, Qilu University of Technology (Shandong Academy of Sciences), Jinan, 250103 China

**Keywords:** 1-Butanol, Non-native hosts, Biofuel production, Synthetic pathways

## Abstract

**Background:**

Owing to the increase in energy consumption, fossil fuel resources are gradually depleting which has led to the growing environmental concerns; therefore, scientists are being urged to produce sustainable and ecofriendly fuels. Thus, there is a growing interest in the generation of biofuels from renewable energy resources using microbial fermentation.

**Main text:**

Butanol is a promising biofuel that can substitute for gasoline; unfortunately, natural microorganisms pose challenges for the economical production of 1-butanol at an industrial scale. The availability of genetic and molecular tools to engineer existing native pathways or create synthetic pathways have made non-native hosts a good choice for the production of 1-butanol from renewable resources. Non-native hosts have several distinct advantages, including using of cost-efficient feedstock, solvent tolerant and reduction of contamination risk. Therefore, engineering non-native hosts to produce biofuels is a promising approach towards achieving sustainability. This paper reviews the currently employed strategies and synthetic biology approaches used to produce 1-butanol in non-native hosts over the past few years. In addition, current challenges faced in using non-native hosts and the possible solutions that can help improve 1-butanol production are also discussed.

**Conclusion:**

Non-native organisms have the potential to realize commercial production of 1- butanol from renewable resources. Future research should focus on substrate utilization, cofactor imbalance, and promoter selection to boost 1-butanol production in non-native hosts. Moreover, the application of robust genetic engineering approaches is required for metabolic engineering of microorganisms to make them industrially feasible for 1-butanol production.

## Background

**Fig. 1:**
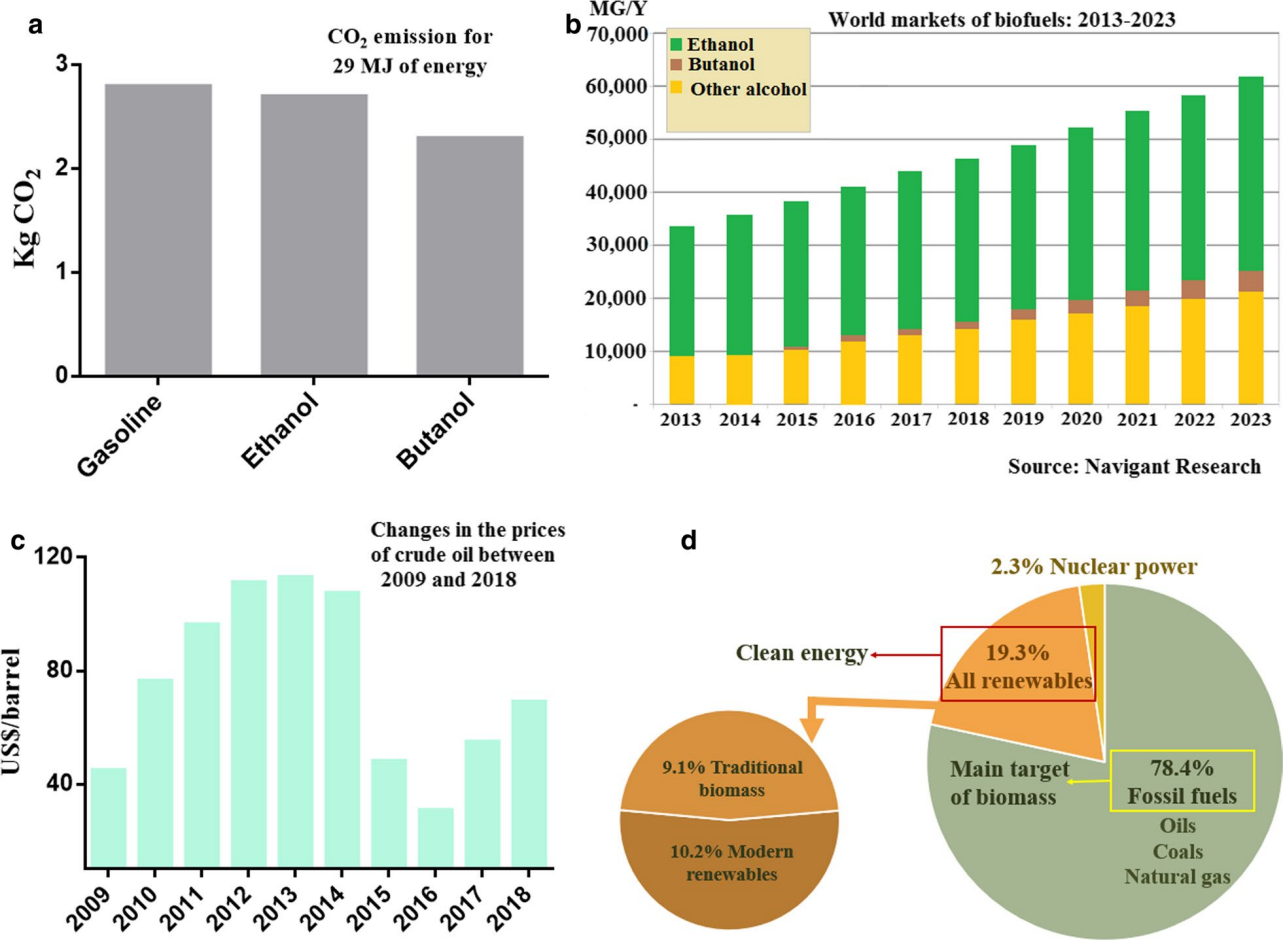
**a** Comparison of CO_2_ emission from different fuels (gasoline, butanol, and ethanol) [5]. **b** Total biofuels production by fuel type around the globe [6]. **c** Fluctuation in the crude oil prices from 2009 to 2018 [10]. **d** World energy consumption by different sources [12]

Butanol is one of the most promising alternative biofuels because it shows better performance than ethanol [1]. Compared to ethanol, butanol has superior physical and chemical properties, such as higher energy density (27 MJ/L vs. 19.6 MJ/L), low water miscibility, low flammability, lesser corrosiveness, and closely resembles to modern-day gasoline (Table 1). Further, butanol can be blended with gasoline in any ratio and used directly in automobile engines without structural modification, besides being transported through the existing pipeline infrastructure [2, 3]. Butanol also has several applications in various chemical industries. It is used as an intermediate in the production of perfumes, paints, polymers (butyl acrylate and butyl methacrylate) and plastics [4]. In addition, the combustion of 1 kg of butanol emits less CO_2_ than that emitted by gasoline; to generate an equivalent amount of energy as that of butanol, a larger amount of ethanol needs to be burned, which results in a higher amount of CO_2_ emission (Fig. 1a) [5]. The global market for butanol was estimated to be 2.8 million tons annually, which is approximately 5 billion USD [6]. Owing to the advantages of butanol over ethanol, there is an increase in the demand for butanol (Fig. 1b). It is expected that the global demands for butanol and other biofuels will surpass 247 billion USD by 2020 [6, 7].

**Table 1:** Comparison of physical and chemical properties of butanol, ethanol, and gasoline [13]

Property	Butanol	Ethanol	Gasoline
Density at 15 °C (kg m^−3^)	810	795	750
Vapour pressure by Reida (kPa)	18.6	16.5	75
Water solubility (mL 100 mL^−1^)	9.1	Miscible	< 0.01
Oxygen content (% vol)	21.6	34.7	< 2.7
Octane number	96	108	95
Calorific value (MJ kg^−1^)	32.5	26.4	43.3

Butanol is produced mostly using petrochemicals (at cost of 7.0–8.4 billion dollars/year) because it is currently cheaper than producing it via biological synthesis [8, 9]. However, the petrochemical synthesis of butanol is very sensitive to crude oil price, which vary considerably (Fig. 1c). Therefore, the petrochemical synthesis of butanol is not a sustainable option that can be continually employed in the near future [10]. Furthermore, the biological production of butanol (biobutanol) is considered as climate neutral. This is because the CO_2_ released from burning biofuels is approximately equal to the carbon sequestered in biomass [11]. Nowadays, approximately 78.4% of the energy is obtained from fossil fuels (Fig. 1d) [12], and with the depletion of fossil fuel resources and its growing environmental concerns, the production of biobutanol through acetone-butanol-ethanol (ABE) fermentation has gained considerable research attention [9]. However, the production of biobutanol via ABE fermentation still faces significant hurdles, which are discussed below.

The production of 1-butanol by ABE fermentation using *Clostridium* spp. faces several challenges. First, this process produces other byproducts such as hydrogen, lactic acid and propionic acid, acetone, ethanol, and isopropanol, which increases the downstream processing cost of 1-butanol purification [14]. These native microorganisms also have some limitations such as slow growth, solvent toxicity, complex nutrient requirements, and complicated life cycle (i.e., spore-production in *Clostridium*) [15, 16]. Furthermore, the genetic manipulation of native producer strains is difficult, and therefore, it is a challenging task to further modify them using different genetic and synthetic biological methods [17]. The current commercial production of 1-butanol through ABE fermentation is based on the fermentation of molasses and starchy materials [18]; the main drawbacks of these fermentation substrates are that the molasses have certain geographical limitations and starchy materials compete with human food. Thus, these natural substrates are not available on a large scale for the production of biofuels [19, 20]. Besides, no solventogenic *Clostridium* spp. utilizes low-cost feedstock such as cellulose and hemicellulose and other organic wastes (e.g., glycerol) directly; however, an exception is *Clostridium pasteurianum* that can use glycerol as a substrate [21, 22]. Therefore, non-native hosts are receiving more attention than those native producers for the production of 1-butanol.

In general, non-native hosts have comparatively simple nutrient requirements, rapid growth rate, known genetic background, and they are easy to manipulate, although these conditions can vary on case to case basis. Further, it is economically feasible to use non-native hosts for large-scale production processes [23–25]. Recent developments in synthetic biology and metabolic engineering have made it possible to easily create a synthetic pathway for the production of biofuels in the non-native hosts [26]. Thus, in recent years, various non-native hosts that cannot produce 1-butanol in their native forms have been equipped with a 1-butanol production pathway. For example, the pathway has been transferred successfully to *Saccharomyces cerevisiae* [27] and *Escherichia coli* [28] owing to their high growth rates. Other microbes such as *Lactobacillus brevis*, *Pseudomonas putida,* and *Bacillus subtilis* were also used for their potentially higher solvent tolerance [29, 30]. *Clostridium cellulovorans* was engineered because of its ability to consume most effective feedstock (cellulose) as a substrate, while a cyanobacterium *Synechococcus elongatus* was used for the direct conversion of CO_2_ into 1-butanol [31, 32]. Thermophilic bacteria *Pyrococcus furiosus* and *Thermoanaerobacterium saccharolyticum* were used for 1-butanol production because of their several distinctive benefits over mesophilic strains. Until now, the most significant advantage of using thermophilic strains is the reduction in contamination risks. All non-native hosts used for 1-butanol production are summarized in Table 2.

**Table 2:** Summary of the non-native hosts used for 1-butanol production

Host	Substrate	Genes overexpressed	Knockout genes	Promoter	1-butanol titer	References
*E. coli*	Glucose and glycerol	*atoB, thl, hbd, crt, ccr, bcd, etfAB* and *adhE2*	Δ*ldhA,* Δ*adhE,* Δ*frdBC,* Δ*fnr,* Δ*pta,* Δ*pntA* and Δ*pflB*	P_L_lacO_1_	552 mg/L	[28]
*E. coli*	Glucose	*thl*, *hbd, crt, bcd, etfAB, adhE1* and *adhE2*	None	P*tac*	1.2 g/L	[36]
*E. coli*	Glucose and glycerol	*atoB*, *hbd, crt, ter, adhE2* and *fdh*	Δ*adhE,* Δ*ldhA,* Δ*frdBC,* Δ*pta,* and Δ*fnr*	P_L_lacO_1_	15 g/L	[37]
*E. coli*	Glycerol and glucose or galactose	*aceEF*-*lpd*^*fbr*^, *atoB*, *hbd, crt, ter, adhE2* and *fdh1*	Δ*ldhA,* Δ*adhE,* Δ*frdBC* and Δ*pta*	P*atoB*	6.8 g/L	[38]
*E. coli*	Glucose and butyrate	*glf, atoDA, acs,* and *adhE2*	Δ*ldhA,* Δ*adhE,* Δ*frdA,* and Δ*pta*	PλP_R_P_L_ and PλP_L_	6.2 g/L	[39]
*E. coli*	Glucose	*glf, atoDA, phaA, hbd, crt, ter* and *adhE2*	Δ*ldhA,* Δ*adhE,* Δ*frdA,* and Δ*pta*	PλP_R_P_*L*_ and PλP_L_	5.5 g/L	[39]
*E. coli*	Cellulose hydrolysate	*glf, atoDA, fdh1, phaA, hbd, crt, ter* and *adhE2*	Δ*ldhA,* Δ*adhE,* Δ*frdA,* and Δ*pta*	P*trc* and PλP_L_	2.8 g/L	[40]
*E. coli*	Cellulose hydrolysate	*glf, phaA, hbd, crt, ter* and *adhE2*	Δ*ldhA,* Δ*adhE,* Δ*frdA,* and Δ*pta*	PλP_R_P_L_ and PλP_L_	5.8 g/L	[40]
*E. coli*	Glucose	*thl, crt, bcd, etfA, etfB, hbd, adhE2* and *ompC*-*tmt*	None	Ppr and P_T7_	320 mg/L	[41]
*E. coli*	Glucose and glycerol	*fdh, pduP, panK, atoB*, *hbd, crt, ter* and *adhE2*	Δ*ldhA,* Δ*adhE,* Δ*frdA,* and Δ*pta*	P_L_lacO_1_	18.3 g/L	[42]
*E. coli*	Glucose	*atoB*, *hbd, crt, ter* and *adhE2*	Δ*adhE,* Δ*frdABCD,* Δ*fdhF,* Δ*mdh,* Δ*yqhD,* Δ*hyc*-*hyp,* Δ*maeB*, Δ*eutE,* Δ*pta,* Δ*ackA,* Δ*pykA*, Δ*ldhA*, Δ*yciAI,* Δ*poxB*, Δ*yagM*	miniP*tac*, P*fdhF,* P_T7_, P*ydfZ* and P*adhE*	20 g/L	[43]
*C. tyrobutyricum*	Glucose	*aad* and *adhE2*	Δ*ack* and Δ*ptb*	P*thl*, P*aad*, and P*ptb*	10 g/L	[44]
*C. tyrobutyricum*		*adhE1* and *adhE2*	Δ*cat1*	P*cat1*	26.2 g/L	[45]
*C. tyrobutyricum*	Mannitol	*aad* and *adhE2*	Δ*ack* and Δ*ptb*	P*thl*, P*aad*, and P*ptb*	16 g/L	[44]
*C. tyrobutyricum*	Glucose and mannitol	*adhE2*	None	P*thl*	6.8 g/L and 20.5 g/L	[46]
*C. tyrobutyricum*	Glucose	*adhE2*	Δ*ack*	P*thl*	16.68 g/L	[47]
*C. tyrobutyricum*	Glucose	*fdh* and *adhE2*	None	P*thl*	12.34 g/L	[48]
*C. tyrobutyricum*	Sucrose and sugarcane juice	*scrA, scrB, scrK* and *adhE2*	Δ*ack*	P*thl*	18.8 g/L	[49]
*C. cellulovorans*	Cellulose	*adhE2*	None	P*thl*	1.42 g/L	[32]
*C. cellulovorans*	Corn cob	*adhE2*	None	P*thl*	3.37 g/L	[50]
*C. cellulovorans*	Deshelled corn cobs	*adhE1* and *ctfAB* and *adc*	None	P*thl*	3.47 g/L	[51]
*C. cellulolyticum*		*atoB, hbd, crt, bcd* and *adhE2*	None	P*thl*	0.12 g/L	[52]
*C. ljungahlii*	Syngas	*thlA, hbd, crt, bcd, adhE,* and *bdhA*	None	P*ptb*	0.15 g/L	[53]
*C. autoethanogenum*	Syngas	*thlA, hbd, crt, bcd* and *etfAB*	None	P*pta*-*ack*	1.54 g/L	[54]
*M. extorquens*	Ethylamine	*ter* and *adhE2*	None	P*mxaF*, Plac, P*meta1_3616*, P*meta1_002*	15.2 mg/L	[55]
*K. pneumonia*	Glycerol	*ter, bdhB,* and *bdhA*	None	P*pk*	15.03 mg/L	[56]
*K. pneumonia*	Glycerol	*Kivd*	None	P*pk*	28.7 mg/L	[56]
*K. pneumonia*	Glycerol	*leuABCD, kivd, adhE1, fdh, gdh and udh*	None	P*tac*	100 mg/L	[57]
*T. saccharolyticum*	Xylose	*thl, hbd, crt, bcd, etfA, etfB and dhE2*	*Δldh, Δpta,* and *Δack*	P*pta*-*ack*, P*gapDH*, and P*cbp*	1.05 g/L	[58]
*P. furiosus*	Maltose	*thl, hbd, crt, ter, bad, bdh, adhA and adhE2*	None	P*porγ*	70 mg/L	[59]
*L. brevis*	Glucose	*crt, bcd, etfB, etfA,* and *hbd*	None	P*bcs*-*operon*	300 mg/L	[29]
*P. putida*	Glucose and glycerol	*thl, hbd, crt, etfAB, bcd, bdhb and adhE1*	None	Plac and P*tac*-lac	44 and 122 mg/L	[30]
*B. subtilis*	Glucose and glycerol	*thl, crt, etfAB, hbd,* and *adhE2*	None	P*hyper*-*spank*	23 and 24 mg/L	[30]
*Synechococcus* 7942	CO_2_	*atoB, hbd, crt, ter* and *adhE2*	None	P_L_lacO_1_ and P*trc*	14.5 mg/L	[31]
*Synechococcus* 7942	CO_2_	*atoB, nphT7, phaB, hbd, crt, phaJ, ter, bldh, yqhD,* and *adhE2*	None	P_L_lacO_1_ and P*trc*	30 mg/L	[60]
*Synechococcus* 7942	CO_2_	*nphT7, phaB, crt, phaJ, ter, pduP* and *yqhD*	None	P_L_lacO_1_ and P*trc*	404 mg/L	[61]
*Synechococcus* 7942	CO_2_	*accase, nphT7, phaB, phaJ, ter, pduP* and *yqhD*	Δ*aldA*	P_L_lacO_1_ and P*trc*	418.7 mg/L	[62]
Synechocystis PCC 6803	CO_2_	*nphT7, phaA, phaB, Slr*0942, *Slr*1192, *crt, phaJ, ccr, fadB, ter, pduP, yjgB, yqhD, mhpF,*	Δ*phaEC,* Δ*ackA,* Δ*slr0168* and Δ*ach*	P*trc*_*20*,_ P*cpc*_*560*_, P*psbA2*	4.8 g/L	[63]
*S. cerevisiae*	Galactose	*atoB, erg10, thl, phaA, phaB, hbd, crt, ccr and adhE2*	None	P*gal1* and P*gal10*	2.5 mg/L	[64]
*S. cerevisiae*	Glucose	*ald6, adh2, erg10, acs, hbd, crt, ter adhE2*	Δ*cit2* and *Δmls1*	P*tef1* and P*pgk*_*1*_	16.3 mg/L	[65]
*S. cerevisiae*	Glucose	*thl, hbd, crt, etfA, etfB, ad, aad,* and *ter*	Δ*gpd1* and Δ*gpd2*	P*gap*	14.5 mg/L	[66]
*S. cerevisiae*	Glucose	*pdh, acs, acl, thl, hbd, crt, ter, bad, bdhB, mhpF, eutE, adhE,* and *adhE2,*	*Δadh1, Δadh4, Δgpd1, Δgpd2, Δcit2* and *Δmls1*		120 mg/L	[67]
*S. cerevisiae*	Glycine and glucose	*goxB, mls1, dal7* and *leu2*	*Δmls1* and *Δleu2*	P*tpi1*	92 mg/L	[68]
*S. cerevisiae*	Glucose	*hom3, hom2, hom6, thr1, thr4, ilv1, cha1, leu4, leu1, adh1, adh2, adh3, adh4, adh5, adh6, bdhB, kivD* and *aro10m*	*Δadh1, Δilv1, Δilv2, Δilv3, Δilv6, Δleu1, Δleu4* and *Δleu9,*	P*tef1* and P*tpi1*	242.8 mg/L	[69]
*S. cerevisiae*	Glucose	*hom3, hom2, hom6, thr1, thr4, leu4, leu1, leu2, leu5, leu9, NFS1, ilv1, cimA, adh7, aro10m and kivD*	*Δadh1*	P*gpd1*	835 mg/L	[70]
*Y. lipolytica*		*EutE, ETR1, ylact1*, *ylact2*, *ylhbd*, *ylcrt, YlGPD, YlMDH, hom6 and YlBDH*s	Δ*YALI*0E17787g	Php4d	123 mg/L	[71]
*A. adeninivorans*	Starch	*thl, hbd, crt, bdh, and adhE2*	Δ*aacp,* Δ*aact,* Δ*aaco,* and Δ*agpd1*	P*aynr1*, P*ayni1*, P*aynt1*, and P*tef1*	20 g/L	[72]

Various heterologous and homogeneous genes expressed/overexpressed, knockout genes in the microbial production of 1-butanol and their corresponding enzymes are as follows: *atoB*, acetyl-CoA acetyltransferase; *thl*, thiolase; *hbd*, 3-hydroxybutyryl-CoA dehydrogenase; *crt*, crotonase; *bcd*, butyryl-CoA dehydrogenase; *etfAB*, electron transfer flavoproteins; *adhE1*, aldehyde/alcohol dehydrogenase; *adhE2*, butyraldehyde-butanol dehydrogenase; *ter*, trans-enoyl-CoA reductase; *fdh*, formate dehydrogenase; *aceF*, dihydrolipoamide acetyltransferase; *lpd*, dihydrolipoamide dehydrogenase; *glf*, glucose facilitator; *atoDA*, acetoacetyl-CoA transferase; *acs*, acetyl-CoA synthetase; *phaA*, β-ketothiolase; *ompC*-TMT, outer-membrane targeted tilapia metallothionein; *pduP*, CoA-acylating aldehyde dehydrogenase; *panK*, pantothenate kinase; *scrA*, sucrose-specific PTS; *scrB*,sucrose-6-phosphate hydrolase or sucrose; *scrK*, fructokinase; *bdh*, butanol dehydrogenase; *bdhA*, butanol dehydrogenase; *bdhB*, Butanol dehydrogenase B; *kivd*, ketoisovalerate decarboxylase; *leuABCD,* 2-isopropylmalate synthase; *adc*, acetoacetate decarboxylase; *gdh*, glucose dehydrogenase; *udh*, pyridine nucleotide transhydrogenase, *bad,* butyraldehyde dehydrogenase; acc*ase*, acetyl-CoA carboxylase; *nphT7*, acetoacetyl CoA synthase; *phaB*, Acetoacetyl-CoA reductase; *phaJ*, acetoacetyl-CoA reductase; *ach*, acetyl-CoA hydrolase; *bldh*, butyraldehyde dehydrogenase; *aldA* and *yqhD*, alcohol dehydrogenase; *erg10*, thiolase; c*cr*, crotonyl-CoA reductase; *adh1*, *adh2*, a*dh3*, *adh4*, a*dh5*, *adh6* and *adh7*, alcohol dehydrogenase; *ald6*, acetaldehyde dehydrogenase; a*d*, aldehyde dehydrogenase; *aad*, aldehyde-alcohol dehydrogenase; *pdh*, pyruvate dehydrogenase; *acs*, acetyl-CoA synthetase; *acl*, citrate lyase; *mhpF*, acetaldehyde dehydrogenase; *eutE*, aldehyde dehydrogenase; *goxB*, glycine oxidase; *leu1* and *leu2*, 3-isopropylmalate dehydrogenase; *leu4* and *leu9*, 2-isopropylmalate synthase, *mls1* and *dal7*, malate synthase; *hom3*, aspartokinase; *hom2*, aspartate-semialdehyde dehydrogenase; *hom6*, homoserine dehydrogenase; *thr1*, homoserine kinase; *thr4*, threonine synthase; *ilv1*/*cha1* threonine deaminase; *kivd*, α-ketoisovalerate decarboxylase; *nfs1*, cysteine desulfurase; *cimA*, citramalate synthase; *ldhA*, lactate dehydrogenase; *frdBC*, fumarate reductase; *pta*, phosphate acetyltransferase; *fnr*, fumarate and nitrate reductase; *pfl*, pyruvate formate lyase; *pntA*, pyridine nucleotide transhydrogenase; *frdA*, fumarate reductase flavoprotein subunit; *ack*, acetate kinase; *cit2*, peroxisomal citrate synthase; *gpd1* and *gpd2*, glycerol-3-phosphate dehydrogenase; *aacp*, peroxisomal acyl-carnitine permease; *aact*, acyl-carnitine transferase; *aaco*, acyl-CoA oxidase

**Fig. 2:**
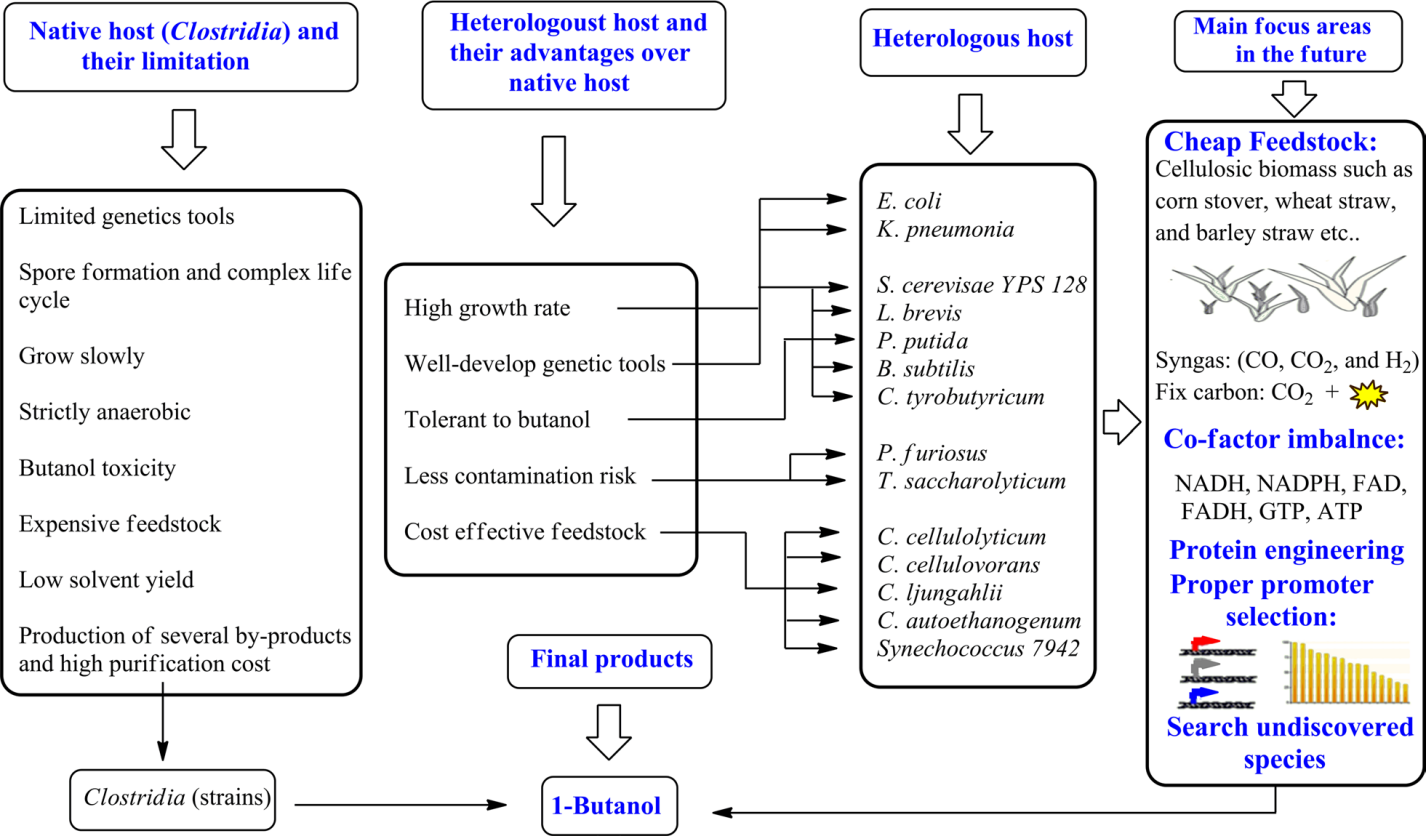
Summary of the limitations faced in the commercial production of 1-butanol using native hosts, and the advantages of non-native hosts along with the microorganisms used for this purpose

The advances and challenges in the engineering of 1-butanol production strains using clostridial species, which are the native 1-butanol producers, have been extensively discussed [8, 16, 33–35]. However, a comprehensive review on the engineering of non-native hosts for 1-butanol production is still absent. Engineering of non-native hosts for 1-butanol production faces special challenges owing to their differences from the clostridial species in preference for carbon source, allocation of metabolic flux, generation of cofactors and other physiological features. To guide the rational design of efficient 1-butanol producers, a focused review presenting a full picture for constructing 1-butanol producers using non-clostridial species is highly desired. Here we have summarized the advantageous characteristics of non-native hosts which include, the ability to use cost-efficient feedstock, high solvent tolerance, and less contamination risk. In addition, different engineered synthetic pathways used for 1-butanol production in non-native hosts have been discussed in detail. This paper also elucidates the current challenges faced in 1-butanol production, as well as the possible solutions that can help to improve the production of 1-butanol, and specific areas that need more focus are indicated (Fig. 2). Therefore, we conclude that this study will prove helpful for researchers to overcome the difficulties in engineering non-native hosts for 1-butanol production and to develop efficient means to meet the growing energy requirements of the world.

## Advantages of non-native host

### Using of alternative carbon sources

#### Cellulose

The cost of the substrate is one of the most significant factors in ABE fermentation, which constitutes approximately 60% of the total cost of the process [73]. Production of 1-butanol through conventional ABE fermentation is based mainly on starchy materials, which are expensive for the fuel market [74]. On the other hand, cellulosic biomass is present abundantly on the biosphere, is less costly, and does not compete with human food. It is also the substrate of choice for producing bulk products by fermentation. Thus, *C. acetobutylicum* was engineered to produce 1-butanol using cellulose as carbon source; however, the engineered strain was unable to utilize cellulose because cellulases are complex enzymes that are difficult to be expressed heterologously [75]. Therefore, Instead of expressing cellulases in native 1-butanol producers, a 1-butanol synthetic pathway was transferred into the cellulolytic microbes, i.e. *C. cellulovorans* and *Clostridium cellulolyticum*, which efficiently converts it into cost-effective advanced biofuels such as 1-butanol [51, 52].

#### Glycerol

Glycerol is a by-product of the biodiesel industry, and the production of 20 kg of biodiesel yields approximately 2 kg of crude glycerol [76]. High abundance and low price make glycerol an attractive feedstock to convert it into valuable products [56]. However, the native 1-butanol producers such as *C. acetobutylicum*, *C. beijerinckii*, and *C. saccharobutylicum* are unable to used glycerol as a sole carbon source [77]. On the other hand, *C. pasteurianum* can utilize glycerol as a carbon source to produce 1-butanol, but due to its complex physiology and limited availability as well as success on the application of genetic tools on it [78], researchers have turned their attention to the non-native producers like *E. coli*. It grows faster than any *Clostridium* species and can use glycerol as a carbon source; therefore, it was engineered for 1-butanol production by transferring the CoA-dependent pathway [28]. Additionally, some other heterotrophs such as *S. cerevisiae*, *P. putida*, *L. brevis,* and *B. subtilis* were also engineered for the production of 1-butanol, using glucose and glycerol as carbon sources. Moreover, another bacterial strain *K. pneumonia* was engineered for the production of 1-butanol [57], which even grows faster and has a similar genetic background to *E. coli* and utilizes glycerol as the sole carbon source.

#### Syngas

**Fig. 3:**
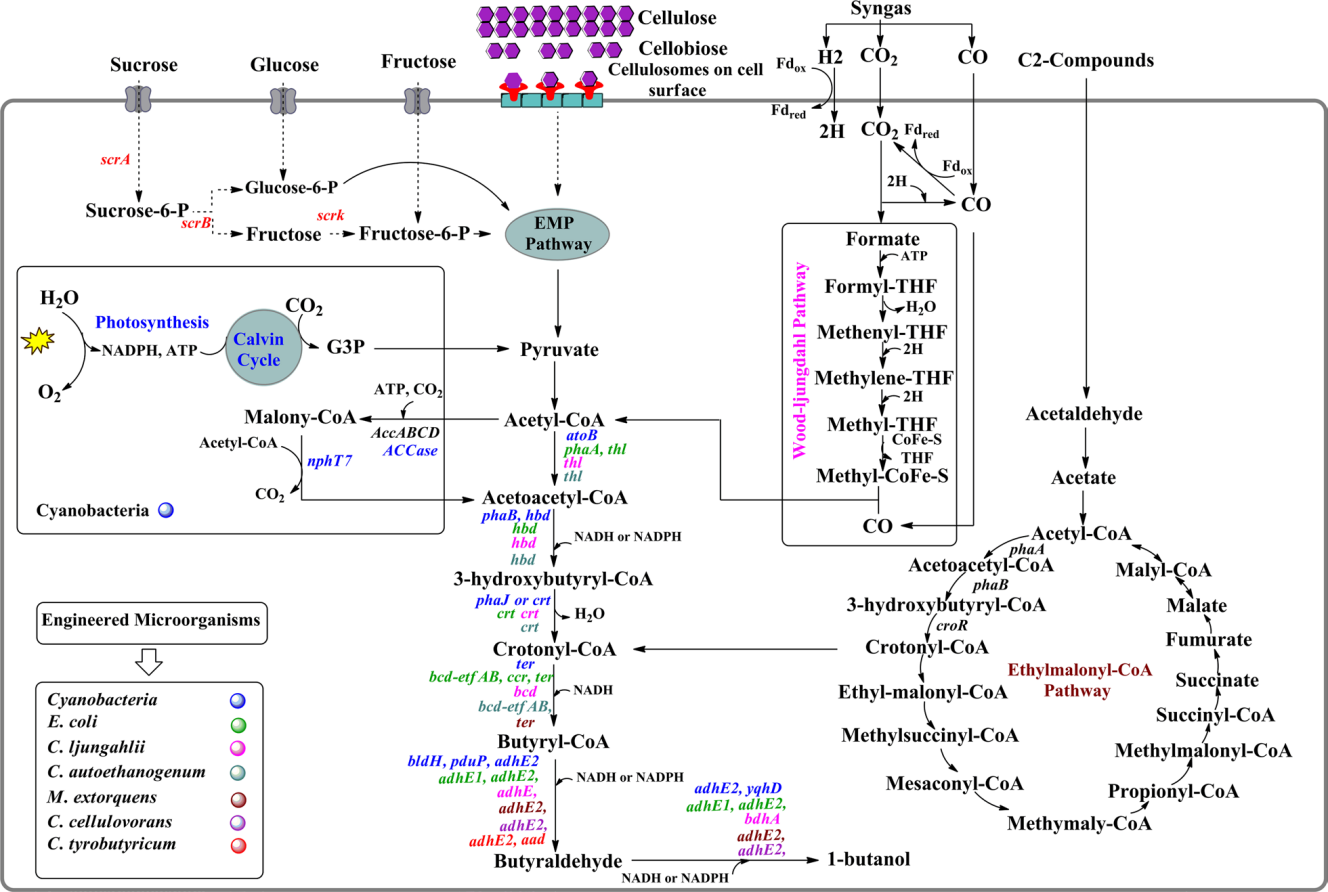
Schematic of 1-butanol production in heterologous hosts from various feedstocks. Colors represent heterologous genes expressed in different hosts

Syngas is a mixture of different gases (CO_2_, CO, and H_2_). It can be obtained from different sources like coal, biomass, and natural gas, which are also found in some industrial waste gases like in steel industries [79]. From an economic point of view, the production of 1-butanol from syngas is preferred over expensive sources such as sugar-based or starchy materials, which do not compete with food crops and concomitantly help in reducing GHG emissions. *Clostridium ljungdahlii* and *Clostridium autoethanogenum* are two acetogenic bacteria that can metabolize such gases via the Wood–Ljungdahl pathway (Fig. 3) [80]. Therefore, *C. ljungdahlii* and *C. autoethanogenum* were engineered to produce cost-effective 1-butanol [53, 54]. Hence, 1-butanol production from syngas is considered to be a promising approach towards economic fuel production.

#### Light and CO_2_

Sunlight and CO_2_ are the most easily available and inexpensive energy and carbon sources on earth. Although the proportion of CO_2_ in the air is low (0 to 0.03%), but the concentration of CO_2_ in the industrial zone is significantly higher. Sometimes in the industrial zone, the level of CO_2_ reached 600 to 700 ppm. Moreover, every year, the concentration of CO_2_ in the atmosphere increases by 2–3 ppm because of the burning of fossil fuels [81]. Cyanobacteria is an excellent microbial cell factory that can utilize solar energy and convert atmospheric CO_2_ to useful products. Additionally, cyanobacteria can grow rapidly, and its genetic manipulation is easy [62]. Owing to its advantages over heterotrophs, cyanobacteria were equipped with a synthetic pathway of 1-butanol, to produce sustainable and eco-friendly biofuels. Recently, a multi-level modular strategy was used to increase the production of 1-butanol in cyanobacterium *Synechocystis* PCC 6803; the engineered strain was able to accumulate the highest titer of 4.8 g/L for 1-butanol with a maximal rate of 302 mg/L/day. It represented the highest 1-butanol titer produced from CO_2_ in cyanobacteria [63]. Thus, cyanobacteria is a promising strain for the production of the cheapest fuels based on solar energy and more sustainable alternatives to the traditional biofuels.

### Resistance to multiple stresses

#### Solvent tolerant

High-value chemicals and biofuels are toxic to native-hosts and as well as to non-native hosts. The toxicity of the solvent is a significant limiting factor that hinders the production of 1-butanol during the fermentation process. Developing a microbial strain with the ability of a solvent tolerance is a difficult task for the researchers [82]. The current metabolic engineering approaches rely mostly on the deletion or overexpression of some functional genes which are useful to some extent, but it does not necessarily achieve the desired level of tolerance. Stress tolerance is a complex mechanism governed through multilayered networks and cannot be achieved through the deletion or overexpression of a single functional gene [83]. In order to increase the 1-butanol tolerance of the native-hosts, various engineering strategies have been employed. However, native-hosts were unable to tolerate more than 2% of 1-butanol concentration [84, 85]. The researchers have taken an interest in isolating 1-butanol tolerant microbial strains that can act as potential alternative hosts for 1-butanol production. Various microbial strains have been identified, which are amendable by genetic engineering approaches and can tolerate much higher 1-butanol concentrations, including *B. subtilis* GRSW2-B1, *L. brevis* (up to 3%), *S. cerevisiae* YPS128 (up to 4%) and *P. putida* (up to 6%) [84, 86–88]. Therefore, considering the solvent tolerant factor, the synthetic 1-butanol pathway was transferred into *L. brevis, P. putida,* and *B. subtilis*, which can grow in the presence of a high amount of 1-butanol [29, 30].

#### Thermophiles

Thermophilic organisms are a fascinating and promising group of microorganisms due to their several distinct advantages over mesophilic organisms when used as a production host. For example, their ability to thrive in extreme conditions such as high temperature lowers the contamination risk and reduces the production cost due to the elimination of the need for sterilization. Additionally, thermophilic conditions provide the opportunity for continuous removal of volatile compounds [89]. Moreover, co-utilization of hexose or pentose is a highly significant trait of biofuel producing microbes. However, some of the mesophilic strains (e.g., mesophilic *Clostridium* sp. and *Zymomonas mobilis*) cannot metabolize two sugars simultaneously due to the mechanism called carbon catabolite repression (CCR) [90, 91]. Interestingly, in many thermophilic organisms, the CCR is absent. In other words, thermophilic organisms (*T. saccharolyticum* JW/SL-YS485, *Thermoanaerobacter* sp. X514) can utilize two sugars (pentose and hexose) simultaneously in an unbiased manner for the production of biofuels [92, 93]. Owing to these advantages, thermophilic and hyper-thermophilic microbes (*T. saccharolyticum* JW/SL-YS485 and *P. furiosus*) were successfully engineered with the synthetic pathway of 1-butanol [58, 59].

## Engineering of non-native hosts for 1-butanol production

### Engineering of CoA-dependent pathway

The clostridial CoA-dependent 1-butanol pathway has been heterologously expressed to produce 1-butanol (Fig. 3). The clostridial 1-butanol pathway begins with the condensation of two molecules of acetyl-CoA to synthesize acetoacetyl-CoA catalyzed by thiolase (*thl*). Acetoacetyl-CoA then undergoes a series of dehydration and reduction to form butyryl-CoA. The genes involved in these enzymatic steps are *hbd*, *crt* and *bcd*-*etf* complex. The final step of clostridial 1-butanol pathway from butyryl-CoA to 1-butanol, is catalyzed by a bifunctional aldehyde/alcohol dehydrogenase, encoded by either *adhE1* or *adhE2*.

Several efforts have been made to develop the heterologous expression of the CoA-dependent 1-butanol pathway in different non-native hosts. However, initial efforts in heterologous expression of the CoA-dependent pathway resulted in the production of a very minute quantity of 1-butanol [28, 31, 53, 60, 64]. One major problem was the *bcd*-*etf* complex enzyme, which expressed poorly in non-native hosts, and this enzyme is also oxygen-sensitive. Second, the initial step of acetyl-CoA condensation is thermodynamically unfavorable. To avoid these problems, alternative enzymes were employed [37, 60, 94]. Solvent tolerant microorganisms, *L. brevis*, *P. putida,* and *B. subtilis* were also engineered for 1-butanol production; however, the engineered strains accumulated lower titers of 1-butanol [29, 30]. A butyric acid producing bacterium, *C. tyrobutyricum* was engineered to produce 1.1 g/L of 1-butanol by the overexpression of *adhE2* gene (Fig. 3), the titers were 3.6-folds, 9-folds and 45-folds higher than the engineered strains of *L. brevis*, *P. putida,* and *B. subtilis*, respectively [44].

Moreover, in order to produce economical 1-butanol, cellulolytic bacteria *C. cellulovorans* and *C. cellulolyticum* were engineered for 1-butanol production. Overexpression of *adhE2* in *C. cellulovorans* resulted in the production of 1-butanol at titers up to 1.42 g/L from cellulose, and 3.37 g/L from pretreated corncob, respectively [32, 50]. Expression of the CoA-dependent pathway led to 1-butanol titers up to 0.12 g/L in *C. cellulolyticum* [52]. To reduce the risk of contamination, the clostridial CoA-dependent pathway was expressed in thermophilic bacteria *P. furiosus* and *T. saccharolyticum* JW/SL-YS485, these engineered strains accumulated 70 mg/L and 1.05 g/L of 1-butanol, respectively [58, 59]. Non-pathogenic and non-conventional *Blastobotrys adeninivorans* haploid yeast was engineered recently, the engineered strain achieved a 1-butanol titer of 20 g/L [72].

The clostridial CoA-dependent pathway was successfully expressed in non-native hosts. However, owing to some limitations of clostridial CoA-dependent pathway such as oxygen-sensitive enzymes, thermodynamically unfavorable steps, and high requirement of reducing power, researchers have explored some alternative 1-butanol synthetic pathways such as Ehrlich pathway, 2-oxoglutarate pathway, and ethylmalonyl-CoA pathway.

### Expression of the Ehrlich pathway for 1-butanol production

**Fig. 4:**
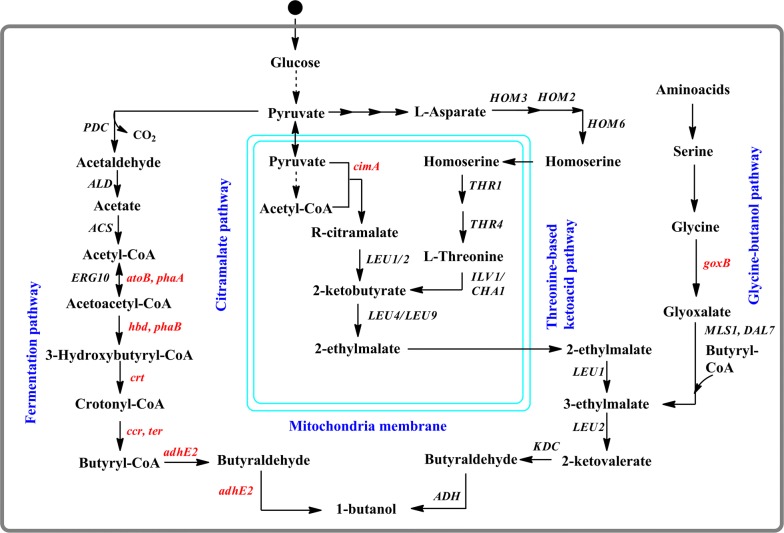
Engineered pathways used for the production of 1-butanol in yeast. Heterologous genes are shown in red and native genes shown in black

1-butanol can also be synthesized via the Ehrlich pathway from 2-ketovalerate (ketoacid) using two reaction steps, by the successive action of a 2-ketoacid decarboxylase (*kivd*) and an alcohol dehydrogenase (*adh*). 2-ketovalerate can be produced from 2-ketobuturate by ketoacid chain elongation process catalyzed by the endogenous enzymes (*LeuABCD*), overexpression of the endogenous enzymes can enhance 1-butanol production. 2-ketobutyrate is an intermediate of amino acid biosynthesis pathway that can be produced via native threonine biosynthetic pathway or the heterologous citramalate pathway (Fig. 4).

Taking advantage of utilizing the amino acid biosynthesis pathway intermediates, the Ehrlich pathway was introduced into *E. coli* for the production of 1-butanol by the expression of heterologous decarboxylase and dehydrogenase [95]. In another study, the citramalate pathway was coupled with the Ehrlich pathway for the production of 1-butanol; the engineered strain produced 0.524 g/L of 1-butanol from glucose [96].

In another study, a novel pathway was created in *S. cerevisiae* by expressing the glycine oxidase (*goxB)* gene from *B. subtilis,* to produce 1-butanol (92 mg/L) using glycine as the substrate via Ehrlich pathway (Fig. 4) [68]. In another approach, an endogenous threonine metabolic pathway was engineered to produce 1-butanol. The overexpression of the endogenous gene related to threonine catabolism and the elimination of other genes affecting the 1-butanol pathway flux generated an engineered strain that produced the highest 1-butanol titer of 242.8 mg/L [69]. Furthermore, to improve the yield of 1-butanol in *S. cerevisiae*, a synergistic approach was applied using the optimization of the endogenous threonine pathway and the expression of the citramalate synthase (CimA) mediated pathway (Fig. 4). The combination of these metabolic engineering pathways with the co-expression of a related gene improved 1-butanol production to 835 mg/L in the final engineered strain under anaerobic conditions, which is almost a sevenfold increase compared to the initial strain [70].

In briefly, 1-butanol has been synthesized successfully using three different routes via the Ehrlich pathway. However, the major bottleneck in this pathway is the production of non-specific alcohol like 1-propanol because of the broad specificity of *kivd* gene. Therefore, to effectively produce 1-butanol via the Ehrlich pathway, significant protein engineering, as well as metabolic engineering efforts, are still required.

### Engineering of ethylmalonyl-CoA pathway

A facultative methylotrophic α-proteobacterium *Methylobacterium extorquens* AM1 can use one-carbon as well as multiple carbon compounds as carbon and energy sources. The *M. extorquens* AM1 is a useful strain in biotechnology as it can produce single-cell proteins, amino acids, poly-β-hydroxybutyrate, and dicarboxylic acids using single carbon compounds [97–99]. It has been shown that during the growth of *M. extorquens* AM1 either using C1 compounds or C2 compounds, a significant metabolic flux occurs through the ethylmalonyl-CoA (EMC) pathway; the EMC pathway is a central metabolic pathway in *M. extorquens* AM1 that converts acetyl-CoA to glyoxylate for reincorporation into the serine cycle during C1 assimilation and replenishes metabolites that either leave the cycle for biosynthetic purposes or are consumed by the tricarboxylic acid (TCA) cycle during C2 assimilation. The EMC pathway provides a continuous supply of crotonyl-CoA, which can be used as a direct precursor for 1-butanol production [100, 101]. Therefore, the native EMC pathway was engineered in *M. extorquens* AM1 for 1-butanol production. Carbon flux was directed from its native EMC pathway towards the 1-butanol pathway by expressing the trans-enoyl-CoA reductase gene from *T. denticola* and the alcohol dehydrogenase gene from *C. acetobutylicum* (Fig. 3). The recombinant strain produced the highest titer of 8.94 mg/L using ethylamine as a substrate. The titer was increased 1.7-fold (15.2 mg/L) by overexpressing the native crotonase [55]. In another study, adaptive laboratory evolution method was used to isolate a 1-butanol tolerant mutant strain (BHBT5 strain) of *M. extorquens* AM1. The adaptive mutant strain BHBT5 produced 1.87-fold (25.5 mg/L) more 1-butanol than the base strain BHB9 after introduction of a plasmid harboring the 1-butanol synthetic pathway [102].

### 2-oxoglutarate pathway for 1-butanol production

In the 2-oxoglutarate pathway, seven enzymatic steps are involved in producing 1-butanol. The resulting set of genes catalyzing seven enzymatic steps are derived from diverse microorganisms. The 2-oxoglutarate pathway uses 2-oxoglutarate intermediate of TCA (try-carboxylic acid) cycle as a starting substrate for 1-butanol production. In the 2-oxoglutarate pathway, first 2-oxoglutarate is converted into 2-hydroxyglutarate by 2-oxoglutarate reductase. In the second reaction, the CoA is transferred from acetyl-CoA to 2-hydroxyglutarate to make 2-hydroxyglutaryl-CoA; this reaction is catalyzed by glutaconate-CoA transferase. In the third reaction, 2-hydroxyglutaryl-CoA is dehydrated into glutaconyl-CoA; this enzymatic step is catalyzed by 2-hydroxyglutaryl-CoA dehydratase. In the fourth reaction, glutaconyl-CoA is decarboxylated into crotonyl-CoA by glutaconyl-CoA decarboxylase. The remaining steps are similar to clostridial pathway. The crotonyl-CoA is reduced to butyryl-CoA by butyryl-CoA dehydrogenase. Finally, the last two steps are successive reductions of butyryl-CoA to butanal and butanal to 1-butanol; the reactions are catalyzed by aldehyde dehydrogenase and alcohol dehydrogenase, respectively [103]. The novel 2-oxoglutarate pathway was introduced into *E. coli* K12, the engineered strain accumulated the highest 1-butanol titer of 85 mg/L by cultivating the cells in bioreactors using glucose as substrate [103]. The titers of 1-butanol obtained through this pathway are still far below from those required for industrial purposes.

### Elimination of the competitive pathways

The strategy of elimination of the competitive pathways has been employed in different non-native hosts, which has successfully increased the titers of 1-butanol by securing the key precursor (like acetyl-CoA) and reducing factor used for 1-butanol synthesis. For example, to increase the flux towards 1-butanol production in *E. coli*, alternative carbon consuming pathways were deleted by the deletion of the *adhE*, *ldhA* and *frdBC* genes, which shifted the carbon flux towards 1-butanol and resulted in twofold increase in 1-butanol production [28]. In another study, the butyrate-conversion strain (BUT-3EA) of *E. coli* was engineered by deleting *ldhA, adhE, frdA,* and *pta* genes and inserting native *atoDA*, *acs* and *Clostridium adhE2* genes. The recombinant strain (BuT-3EA) was able to accumulate a titer of 6.2 g/L of 1-butanol using glucose and butyrate as a carbon sources [39], the titers were 1.5-fold higher than the previously engineered strain using glycerol and butyrate as carbon sources [104].

Similarly, Yu et al. deleted *ack* (encoding acetate kinase) in *C. tyrobutyricum* to reduce the acetyl-CoA conversion to acetate. The deletion of *ack* led to a ninefold increase in the titer of 1-butanol [44]. In another study, different replicons from various microorganisms were evaluated to overexpress the *C. acetobutylicum adhE2* gene in *C. tyrobutyricum*. The recombinant strain (without any gene deletion) was able to produce the highest titers of 6.8 g/L and 20.5 g/L of 1-butanol, using glucose and mannitol as the carbon sources, respectively; while the ACKKO-*adhE2* mutant (*∆ack*-*adhE2*) accumulated 16.68 g/L of 1-butanol (2.5-fold increase) using glucose as the carbon source [46, 47]. Recently, Zhang et al. successfully replaced the *cat1* (butyrate: acetate coenzyme A transferase) with *adhE2*, the mutant Δ*cat1*::*adhE2* strain produced 26.2 g/L 1-butanol in batch fermentation. This is the highest 1-butanol production that has ever been reported in batch fermentation [45].

Sakuragi et al. deleted *GPD1* and *GPD2* (encoding glycerol- 3-phosphate dehydrogenases) genes in *S. cerevisiae* that compete with 1-butanol production by utilizing the same intermediate metabolites, the deletion of the *GPD1* and *GPD2* genes resulted in approximately 1.3-fold increase in 1-butanol production [66]. Additionally, the combined deletion of the *ADH1*, *ADH4*, *GPD1,* and *GPD2* genes involved in ethanol and glycerol biosynthesis in *S. cerevisiae*, redirected the carbon flux towards acetyl-CoA, resulting in a fourfold increase in 1-butanol production [67]. Moreover, deletion of *CIT2* (encoding citrate synthase) and overexpression of aldehyde dehydrogenase (*Ald6*), alcohol dehydrogenase (*Adh2*), and acetyl-CoA synthetase mutant (*Acs*^L641P^) to enhance the carbon flux towards cytosolic acetyl-CoA, also led to a 2.5-fold increase in 1-butanol titer [65].

### Engineering of rate-limiting steps in 1-butanol production

Metabolomics is a comprehensive study of metabolites in a biological sample. In metabolic engineering, a metabolomics approach is an excellent strategy to search for the rate-limiting step in a biosynthetic pathway. Using metabolomics strategy, Noguchi et al. revealed that the reduction reaction of butanoyl-CoA to butanal catalyzed by acylating aldehyde dehydrogenase (*PduP*) is a possible rate-limiting step in the 1-butanol pathway in *Synechococcus elongatus* [105]. Therefore, by enhancing the activity of *PduP* and inserting the gene encoding the subunit of the ACCase from *Yarrowia lipolytica* into the *aldA* (encodes for alcohol dehydrogenase) site, *S. elongatus* recombinant strain DC11 was able to accumulate 418.7 mg/L of 1-butanol, which is almost 1.4-fold more than its parent strain BUOHSE after 6 days of fermentation [62].

Similarly, Ohtake et al. also used a metabolomics approach to determine the bottleneck in the engineered pathway of 1-butanol. They discovered that the deletion of the *pta* gene (encoding phosphate acetyltransferase) resulted in pathway imbalance as it prevents the release of CoA; they identified the *adhE2*-mediated reaction (conversion of butyryl-CoA to butyryl-dehyde) as the rate-limiting step. Thus, a simultaneous balancing of the CoA and enhancing the activity of *adhE2* increased the titer of 1-butanol up to 1.3-fold (*E. coli* JCL299FT strain) compared with its parent strain JCL299F under anaerobic conditions [42]. These results indicate that engineering the rate-limiting step has a significant effect in improving the production of 1-butanol.

### Comparison of the different pathways of 1-butanol

Different synthetic pathways aforementioned were used for the production of 1-butanol. In these engineered pathways, clostridial CoA-dependent 1-butanol pathway is one of the most well-known pathway used for 1-butanol production in the heterologous hosts. One of the major advantages could be that the clostridial CoA-dependent pathway is a natural pathway, therefore there is no need to design new reaction or to mine for novel enzymes like 2-oxoglutarate pathway. However, the major limitation of the clostridial CoA-dependent 1-butanol pathway; is the length (six enzymatic steps) of the pathway, which needs more enzymes to be expressed and thus, the cell takes a greater metabolic burden compared with Ehrlich pathway. Another bottleneck in the reconstruction of the clostridial CoA-dependent pathway in the heterologous hosts is the poor expression of oxygen susceptible nature of enzymes [61].

Recently, 2-oxoglutarate pathway of 1-butanol was employed for the production of 1-butanol in *E. coli* strain. The key advantage of 2-oxoglutarate pathway is the thermodynamically favorable first step to avoid unfavorable condensation of two molecules of acetyl-CoA in comparison to the clostridial pathway [37, 103]. However, using 2-oxoglutarate pathway to produce 1-butanol requires the carboxylation of one phosphoenolpyruvate to oxaloacetate and the activation of 2-hydroxyglutarate to 2-hydroxyglutarylCoA using acetyl-CoA as a donor. These steps need extra energy, which makes the 2-oxoglutarate pathway less advantageous in comparison to clostridial pathway [103].

Another pathway producing 1-butanol is the Ehrlich pathway. The advantages of the Ehrlich pathway is the requirement of only two heterologous enzymatic steps in comparison with 2-oxoglutarate pathway and clostridial pathway, which consist of seven and six enzymatic steps, respectively. Additional advantage of Ehrlich pathway is that it does not need high amount of reducing factor like 2-oxoglutarate and clostridial pathways. Another major advantage of the Ehrlich pathway over CoA-dependent pathway is that it circumvents the production of toxic metabolites, especially CoA-dependent intermediates [95]. However, the production of 1-butanol through the Ehrlich pathway is only 0.8/L (Table 3), which is about a 30-fold lower than CoA-dependent pathway of 1-butanol. One reason of the lower production of 1-butanol via the Ehrlich pathway is the divergence of 2-ketobutyrate (key precursor of 1-butanol) into the isoleucine biosynthetic pathway. Additionally, some of the enzymes involves in threonine biosynthetic pathway are inhibited by their products. Another bottleneck of the Ehrlich pathway is the production of a mixture of alcohols (1-propanol, 2-methyl 1-butanol, isobutanol, 3-methyl 1-butanol and 2-phenyl ethanol), due to the broad substrate specificity of the *kivd* gene, and thus reduces the overall amount of 1-butanol production [106]. However, it should be noted that most of these alcohols are ideal alternatives to traditional gasoline and are suitable for engine fuel usage.

**Table 3:** Pathways expressed in non-native hosts for 1-butanol production

Pathway	Host	Titer (g/L)	References
CoA-dependent pathway	*C. tyrobutyricum*	26.2	[45]
	*A. adeninivorans*	20	[72]
	*E. coli*	20	[43]
	*Synechocystis* PCC 6803	4.8	[63]
	*C. cellulovorans*	3.37	[50]
	*C. autoethanogenum*	1.54	[54]
	*T. saccharolyticum*	1.05	[58]
	*L. brevis*	0.300	[29]
	*C. ljungahlii*	0.15	[53]
	*P. putida*	0.122	[30]
	*C. cellulolyticum*	0.12	[52]
	*P. furiosus*	0.070	[59]
	*B. subtilis*	0.024	[30]
Ethylmalonyl-CoA pathway	*M. extorquens*	0.015	[55]
Ehrlich pathway	*S. cerevisiae*	0.835	[70]
	*E. coli*	0.80	[95]
	*K. pneumonia*	0.1	[57]
2-oxoglutarate pathway	*E. coli*	0.085	[103]

In summary, the results above demonstrate that the clostridial CoA-dependent pathway performs better among all of the 1-butanol pathways expressed in non-native hosts. Recently, a record high 1-butanol production was achieved by the recombinant strain of *C. tyrobutyricum* (Table 3) via the CoA-dependent pathway [45], which is higher than that from most of the native 1-butanol producers. Additionally, the engineered strain of *E. coli* produced 1-butanol to a titer (20 g/L) with yield of 34% (w/w 83% of theoretical yield) in batch fermentation [43], which is at comparable level with 21 g/L of 1-butanol accumulated by adapted native producer *C. acetobutylicum* strain JB200 [107]. However, some of the non-native hosts produced less amount of 1-butanol. Nevertheless, a considerable room still exists in modifying the existing biosynthetic pathways for 1-butanol production or in the search for the alternative 1-butanol pathways, which are more suitable for expression in non-native hosts.

## Current challenges and solutions for 1-butanol production using non-native hosts

### Cofactor imbalance

The introduction of a non-native pathway into a microorganism can cause a cofactor imbalance, which results in a metabolic burden on the cells. Therefore, various cofactor engineering strategies have been developed to regulate the redox balance and improve the yield and efficiency of microbial cell factories [108]. To maintain redox balance in the 1-butanol synthetic pathway, four strategies were employed, i.e., regulation of endogenous cofactor systems [37], heterologous cofactor regeneration systems [48], modifying cofactor preferences [60, 109], and synthetic cofactor [32, 49, 110]. For example, endogenous cofactor regeneration strategy and acetyl-CoA were coupled as driving forces to direct the carbon flux in *E. coli*, through which 1-butanol production was improved to a level comparable to that produced by the *Clostridium* species, with a 1-butanol titer of 30 g/L and increasing the yield to 70% of the theoretical level [37]. However, in the absence of cofactor regeneration strategy, only the modified metabolic pathway achieved about 10% of the theoretical yield [37]. In another approach, endogenous cofactor regeneration and heterologous cofactor regeneration strategies were employed simultaneously in recombinant *E. coli* by activating *pdh* gene anaerobically that encodes pyruvate dehydrogenase complex and with the expression of *S. cerevisiae* NAD^+^-dependent formate dehydrogenase (*fdh1*), which efficiently enhanced the titer of 1-butanol [38]. These results revealed that cofactor engineering is an effective strategy to increase the metabolic flux towards desired products.

### Choice of genes

Various recombinant pathways have been constructed for the production of 1-butanol in different microbes. However, the major hurdle facing the non-native hosts is the poor expression of heterologous enzymes. For example, the clostridial 1-butanol pathway is the most employed pathway in the non-native hosts. As aforementioned that the major problem of the clostridial 1-butanol pathway is the poor expression of the oxygen-sensitive enzyme complex (*bcd*-*etf*) in non-native hosts and its need for ferredoxin as an additional redox partner. These factors hampered the enzyme activity in the non-native hosts, and thus the expression of *bcd*-*etf* complex led towards the production of a very minute quantity of 1-butanol in the non-native hosts. To circumvent the problem of poor expression of enzymes, alternative enzymes were proposed to improve the titers. For example, *bcd*-*etf* complex was replaced by *ter* (encoding trans-enoyl-CoA reductase) gene from *Treponema denticola*, this gene replacement led to an 18-fold increase in titer of 1-butanol in *E. coli* after 24 h of fermentation [37, 111]. In another study, the production of 1-butanol was increased more than threefold by replacing the *thl* gene with *E. coli atoB* gene due to its higher specific activity for acetyl-CoA than *thl* [28]. In *S. cerevisiae*, 1-butanol production was improved by replacing *ccr* (*S. collinus*) with *ter* gene [65]. Similarly, in photosynthetic bacteria such as cyanobacteria, which generates oxygen, the oxygen-sensitive enzymes created various problems in 1-butanol pathway. To solve the oxygen-sensitivity problem of *S. elongatus* PCC 7942, the *bldh* was substituted by oxygen-tolerant CoA-acylating aldehyde dehydrogenase (encoded by *pdup*) from *Salmonella enterica* in the 1-butanol synthetic pathway. The modified pathway resulted in 1-butanol production to a cumulative titer of 404 mg/L, exceeding the parent strain expressing *bldh* by 20-fold [61]. These studies demonstrate that the choice of gene is one of the major and deciding factors for enhancing 1-butanol production in the non-native hosts.

### Pathway imbalance

Heterologous pathways are usually constructed by the combination of multiple genes from different species. However, transferring the enzymatic pathways from one organism to another often cause a loss of the regulation, and thus imbalances occur in gene expression as well as enzymatic activity [112]. Therefore, several approaches have been developed through which pathway can be balanced, such as promoter engineering, RBS engineering and gene copy number [94, 113, 114]. Promoters are significant genetic elements that play an important role in controlling gene expression in both the heterologous and endogenous pathways. Uncontrolled gene expression in a heterologous host causes a metabolic burden on the cell, and results in the reduction of cellular biomolecules that are important for the growth of the cell itself. Further, it also generates unwanted physiological changes [115]. Therefore, proper promoter selection is essential for smooth growth and high productivity of the desired products. The synthetic pathways should be balanced to direct the flux towards the product. In many cases, lowering the expression of specific enzymes in the pathways is beneficial to achieve higher productivity [116, 117]. This is mostly because some synthetic pathways may accumulate toxic intermediates, which would inhibit cell growth and productivity. Reversibility is another major obstacle in metabolic pathways. Bonds-Watts et al. employed a two-promoter system for 1-butanol production (strong promoter T7 and weak arabinose promoter) to shift the overall equilibrium towards 1-butanol. This shows the importance of appropriate promoter selection in the case of reversibility [94]. In another approach, in *S. cerevisiae* the production of 1-butanol was increased 100-fold by balancing 1-butanol pathway enzymes through a set of tunable copy number plasmids (which were dependent on antibiotic concentration) [118]. These studies showed that balancing of enzymes in the 1-butanol pathway has a significant role in achieving a high titer of biofuels.

## Conclusion and future perspectives

Compared with ethanol, 1-butanol is a superior biofuel and is gaining global research attention in several industrial fields. Native microorganisms are difficult to manipulate for 1-butanol production; however, in recent years, the metabolic engineering of a heterologous host for 1-butanol production has received an enormous amount of research interest with the rapid progress in gene editing and synthetic biology technologies. Various non-native organisms are being equipped with metabolic pathways for converting renewable resources into 1-butanol. Approaches including the use of exogenous enzymes with higher activity, and the removal of competitive pathways have helped in achieving further improvements in 1-butanol production. However, these non-native hosts have limitations such as issues with feedstock, contamination, and toxicity. To overcome these limitations, various microorganisms such as cellulolytic and photosynthetic bacteria, thermophiles, and solvent-tolerant organisms were engineered to produce 1-butanol. However, despite the high potential of these microorganisms, 1-butanol production using these microorganisms still has a far lower titer that is required for commercial production. Therefore, future research should focus on cofactor imbalance, proper promoter and gene selection, product toxicity, and enhancing substrate utilization in order to boost 1-butanol production. Additionally, improving the activity of key metabolic enzymes involved in a 1-butanol pathway by protein engineering is also a promising approach to enhance 1-butanol production. Moreover, lignocellulose or CO_2_ utilizing microbes are much difficult to engineer than *E. coli*; therefore, new genetic tools are required to make them industrially feasible.

## Data Availability

Data sharing is not applicable to this article as no datasets were generated or analysed during the current study.

## Abbreviations

ABE: Acetone–butanol–ethanol
RBS: Ribosome-binding sites
ROS: Reactive oxygen species
GHG: Greenhouse gas
EMC: Ethylmalonyl-CoA
ATP: Adenosine triphosphate
NAD^+^: Oxidized nicotinamide adenine dinucleotide
NADH: Reduced nicotinamide adenine dinucleotide
NADPH: Reduced nicotinamide adenine dinucleotide phosphate
CoA: Coenzyme A
